# A191 THE NEURAL SIGNAL, CALCITONIN GENE-RELATED PEPTIDE (CGRG) ENHANCES A REGULATORY PHENOTYPE IN THE HUMAN IL-4-TREATED MACROPHAGE

**DOI:** 10.1093/jcag/gwad061.191

**Published:** 2024-02-14

**Authors:** B E Callejas Pina, J A Sousa, K Flannigan, A Wang, S Li, S Rajeev, R Panaccione, D McKay

**Affiliations:** Physiology and Pharmacology, University of Calgary, Calgary, AB, Canada; University of Calgary, Calgary, AB, Canada; Physiology and Pharmacology, University of Calgary, Calgary, AB, Canada; Physiology and Pharmacology, University of Calgary, Calgary, AB, Canada; Physiology and Pharmacology, University of Calgary, Calgary, AB, Canada; Physiology and Pharmacology, University of Calgary, Calgary, AB, Canada; Physiology and Pharmacology, University of Calgary, Calgary, AB, Canada; Physiology and Pharmacology, University of Calgary, Calgary, AB, Canada

## Abstract

**Background:**

Interlukin-4 activated human macrophages (M(IL4) promote epithelial wound healing and exert an anti-colitic effect in a murine model. Blood monocyte-derived M(IL4)s from healthy donors and individuals with Crohn’s disease had increased mRNA expression of the calcitonin gene-related peptide (CGRP) receptor chain, RAMP1, raising the issue of neural modulation of the M(IL4)s reparative function.

**Aims:**

To determine if CGRP-RAMP1 signalling in human IL-4 treated macrophages enhances the anti-colitic phenotype.

**Methods:**

Peripheral blood mononuclear cells from healthy volunteers (HD) and individuals with Crohn’s disease were cultured on plastic (2h, 37°C) and non-adherent cells removed. The adherent cells were cultured with recombinant hM-CSF (10 ng/ml) for 7 days. The resultant macrophages (2.5×10^5^) were differentiated with IL-4 (48h 10 ng/mL). In other cells, CGRP (10 nM) was added 24h after IL-4 and phenotype assessed by qPCR. *Rag1*^*-/-*^ mice were treated with M(IL4)±CGRP (1x10^6^ i.p.): 48h later, colitis was induced by oxazolone. FITC dextran was used to evaluate epithelial barrier integrity. A wound healing assay was conducted using CaCo2 epithelial cells and conditioned medium (CM,1:2 dilution) from the different treatments. Prostaglandin D_2_ production was evaluated by ELISA and cyclooxygenase (COX)-1 and -2 activity inhibited using SC560 and indomethacin.

**Results:**

Compared to non-treated macrophages (M(0)), M(IL4)s from HD had increased RAMP1 and CLR mRNA. M(IL4)s treated with CGRP showed enhanced expression of CD206, CCL26, RAMP1 and CCL18. When delivered systemically to oxazolone-treated *rag1*^*-/-*^ mice, M(IL4,CGRP) had an anti-colitic effect superior to M(IL4)s from the same healthy blood donor. Use of the the human colon CaCo2 cell *in vitro* wounding model revealed that CM from M(IL4,CGRP) had increased amounts of TGFb and increased wound-healing capacity compared to matched M(IL4)-CM. Moreover, M(IL4,CGRP)s displayed increased COX-1 and PGD_2_. CM from M(IL4,CGRP)s treated with indomethacin or SC-560 to inhibit COX1 activity failed to promote repair of wounded CaCo2 cell monolayers.

**Conclusions:**

Neuronal input is revealed as an important local modifier capable of boosting the anti-colitic effect of autologous M(IL4) transfer to treat enteric inflammation.

**Funding Agencies:**

CCCHelmsley Charity

